# Supplementary material for: Salmonella Infection in Turtles: A Risk for Staff Involved in Wildlife Management?
Source: Animals (Basel). 2021 May 24;11(6):1529. doi: 10.3390/ani11061529 (PMC8225080; doi:10.3390/ani11061529)
Supplement: Supplementary file 1 [file animals-11-01529-s001.zip › animals-1205883-supplementary.pdf]

Table 1S. Gender, presumed age and morphometric measures of turtles tested in the study

ID	Specie	Gender	Presumed age	Body weight	Shell length (mm)	Shell width (mm)	Shell height (mm)
T1	*T. h. hermanni*	F	More than 10 years	814	161	106	62
T2	*T. h. hermanni*	M	More than 10 years	565	133	106	62
T3	*T. h. hermanni*	F	More than 10 years	837	151	111	71
T4	*T. h. hermanni*	F	More than 8 years	579	135	111	60
T5	*T. h. hermanni*	F	More than 10 years	1039	163	126	72
T6	*T. h. hermanni*	F	More than 8 years	561	139	104	54
T7	*T. h. boettgeri*	F	More than 10 years	914	160	119	73
T8	*T. h. hermanni*	M	More than 8 years	548	133	105	63
T9	*T. h. hermanni*	M	More than 7 years	396	127	94	57
T10	*T. h. hermanni*	F	More than 10 years	808	154	122	60
T11	*T. h. hermanni*	M	About 7 years	337	113	89	43
T12	*T. h. hermanni*	F	More than 7 years	469	138	106	68
T13	*T. h. hermanni*	F	About 6 years	304	111	85	47
T14	*T. h. hermanni*	F	About 7 years	356	113	93	44
T15	*T. h. hermanni*	M	More than 7 years	408	122	98	54
T16	*T. h. hermanni*	F	More than 10 years	900	169	127	66
T17	*T. h. hermanni*	F	More than 10 years	773	153	114	67
T18	*T. h. hermanni*	M	About 7 years	375	118	92	47
T19	*T. h. hermanni*	F	About 10 years	547	141	100	60
T20	*T. h. hermanni*	M	About 10 years	559	145	103	64
T21	*Hybrid of T. h. hermanni*	M	More than 10 years	581	143	118	56
T22	*Hybrid of T. h. hermanni*	F	More than 10 years	585	161	112	63
T23	*T. h. hermanni*	F	More than 7 years	430	121	102	52
T24	*T. h. hermanni*	F	More than 7 years	413	122	152	65
T25	*Hybrid of T. h. hermanni*	M	More than 10 years	979	167	124	66
T26	*T. h. hermanni*	F	More than 7 years	411	125	105	58

T27	*T. h. hermanni*	M	More than 10 years	517	133	113	63
T28	*N.G.A.*	M	About 7 years	440	107	82	56
T29	*N.G.A.*	F	More than 7 years	430	125	102	62
T30	*T. h. boettgeri*	M	More than 7 years	410	133	111	56
T31	*T. h. hermanni*	M	About 7 years	284	113	93	66
T32	*N.G.A.*	M	More than 8 years	445	123	95	55
T33	*N.G.A.*	M	More than 7 years	378	123	100	53
T34	*N.G.A.*	M	More than 8 years	480	142	107	72
T35	*Hybrid of T. h. hermanni*	M	More than 10 years	805	213	193	96
T36	*T. h. boettgeri*	M	More than 10 years	613	151	105	65
T37	*T. h. hermanni*	M	More than 10 years	493	142	109	61
T38	*T. h. boettgeri*	M	More than 7 years	439	123	111	53
T39	*T. h. hermanni*	M	More than 10 years	541	140	123	52
T40	*T. h. hermanni*	F	More than 10 years	617	141	112	76
T41	*N.G.A.*	M	More than 8 years	442	132	116	60
T42	*N.G.A.*	F	More than 10 years	1100	173	141	66
T43	*T. h. boettgeri*	M	More than 10 years	844	160	126	71
T44	*T. marginata*	M	More than 10 years	1043	158	133	101
T45	*T. h. boettgeri*	M	More than 10 years	1138	170	144	73
T46	*N.G.A.*	M	More than 7 years	407	118	90	62
T48	*N.G.A.*	F	More than 10 years	670	146	106	75
T49	*N.G.A.*	F	More than 10 years	755	153	131	76
T50	*N.G.A.*	F	More than 7 years	458	138	103	64
T51	*T. graeca*	F	More than 10 years	2078	196	152	80
T52	*T. h. boettgeri*	F	More than 10 years	1185	172	152	84
T53	*Hybrid of T. h. hermanni*	F	About 6 years	240	100	90	44
T54	*N.G.A.*	M	About 6 years	271	103	76	59
T55	*T. graeca*	M	More than 8 years	487	130	89	66

T56	*Hybrid of T. h. hermanni*	M	More than 8 years	411	122	100	54
T57	*N.G.A.*	F	More than 10 years	827	162	128	68
T58	*T. marginata*	F	More than 10 years	4122	317	138	113
T59	*Hybrid of T. h. hermanni*	M	More than 8 years	370	132	108	51
T61	*T. h. hermanni*	M	More than 7 years	361	121	103	56
T62	*T. h. hermanni*	F	More than 10 years	705	141	124	71
T63	*T. h. hermanni*	F	More than 10 years	1317	189	141	77
T64	*T. h. hermanni*	M	About 7 years	259	110	81	58
T65	*T. h. hermanni*	M	More than 8 years	496	134	106	58
T66	*T. h. hermanni*	F	More than 10 years	703	140	111	61
T67	*N.G.A.*	M	More than 8 years	490	135	114	53
T68	*T. h. boettgeri*	M	More than 8 years	392	123	93	52
T69	*T. h. hermanni*	M	More than 8 years	586	145	110	59
T70	*N.G.A.*	F	More than 10 years	1326	181	146	81
T71	*N.G.A.*	F	More than 10 years	1213	183	146	79

*N.G.A. = not genetically analysed.

** Straight length

*** Straight width

Table S2. – List of molecular markers used to genotype captive *T. hermanni* in this study. For each locus, the target DNA analyzed (Type), the length of the fragments amplified (bp), the primer pairs used (including sequences and labels) and their reference (Ref), and the thermal PCR profile have been reported.

Locus	Type	bp	Primer	Primer sequences(5'-3')	Ref	Thermal profile
cytb	mtDNA	372	mt-c-For2<br>mt-E-Rev2	TGAGGVCARATATCATTYTGAG<br>GCRAATARRAAGTATCATTCTGG	[14]	94°C (5 min), 35 x [94°C (30 s), 49°C (45 s), 72°C (90 s)], 72°C (5 min)
Ther20	nuDNA	130-140 *(109-169)*	Ther20*<br>Ther20	FAM-AGGAGAAAAGGGCCTGTCTG<br>GTTGCTGTCTTCCTTGCCTC	[10]	94°C (3 min), 30 x [94°C (30 s), 58°C (60s), 72°C (60 s)], 72°C (5 min)
Ther51	nuDNA	177-189 *(181-205)*	Ther51*<br>Ther51	HEX-AAGGTGGAAGACTCAAAACTGC<br>AGAGCCCTTAAATTCCAGTTAGA	[10]	94°C (3 min), 30 x [94°C (30 s), 58°C (60s), 72°C (60 s)], 72°C (5 min)
Ther94	nuDNA	94-114 *(94-150)*	Ther94*<br>Ther94	HEX-GGGAATGACTTGAGCACTGG<br>AGTTATCAGCATATCTGTAAAGACTGA	[10]	94°C (3 min), 30 x [94°C (30 s), 56°C (60s), 72°C (60 s)], 72°C (5 min)
Test71	nuDNA	124-128	Ther71*<br>Ther71	FAM-GATTGTGGTCACATATAGAGGAGG	[34]	94°C (3 min), 30 x [94°C (30 s), 56°C (60s), 72°C (60 s)], 72°C (5 min)

		(126-130)		TGTTGTACTTAGCTGTTCTGA TCTATT		
Gal263	nuDNA	72-110 *(80-164)*	Gal263* Gal263	HEX-GGGAAAGTACTATTTCCAGA GCTGG GCTGAGGCTAGCTAATTTTTA TGT	[33]	94°C (3 min), 30 x [94°C (30 s), 50°C (60s), 72°C (60 s)], 72°C (5 min)
Test56	nuDNA	193-201 *(203-205)*	Ther56* Ther56	FAM-GATATGCAGGCAAACAGGCT CAGGAATCTGTGCATGATTGA	[34]	94°C (3 min), 30 x [94°C (30 s), 56°C (60s), 72°C (60 s)], 72°C (5 min)
Test10	nuDNA	176-228 *(194-228)*	Ther10* Ther10	HEX-AGACTCTCTGTGATGGTAATA GCA GATTTTCATTGGCATATAAGA CACA	[34]	94°C (3 min), 30 x [94°C (30 s), 53°C (60s), 72°C (60 s)], 72°C (5 min)
Test76	nuDNA	116-118 *(116-118)*	Ther76* Ther76	FAM-GAATTCTAACTTTTCTCTGTG GAGC TCTTATTGCATATCTGAGTAC AGAAGA	[34]	94°C (3 min), 30 x [94°C (30 s), 58°C (60s), 72°C (60 s)], 72°C (5 min)
